# Response inhibition in children with different subtypes/presentations of attention deficit hyperactivity disorder: A near-infrared spectroscopy study

**DOI:** 10.3389/fnins.2023.1119289

**Published:** 2023-03-02

**Authors:** Yike Zhu, Siqi Liu, Fan Zhang, Yongying Ren, Tingyu Zhang, Jing Sun, Xin Wang, Lin Wang, Jian Yang

**Affiliations:** ^1^Center of Children’s Healthcare, Children’s Hospital Affiliated to Capital Institute of Pediatrics, Beijing, China; ^2^Department of Neurology, Children’s Hospital Affiliated to Capital Institute of Pediatrics, Beijing, China; ^3^Department of Rehabilitation Medicine, The Seventh Affiliated Hospital, Sun Yat-sen University, Shenzhen, China

**Keywords:** functional near infrared spectroscopy (fNIRS), ADHD, subtype, children, response inhibition, PFC

## Abstract

**Background:**

Executive dysfunction in children with attention deficit hyperactivity disorder (ADHD) is thought to be closely related to the prefrontal cortex (PFC). However, there is controversy over the activation of the PFC in children with ADHD. Differences could be related to the subtype. Meanwhile, no study to date has used functional near-infrared spectroscopy (fNIRS) to explore the differences between subtypes. Thus, this study aimed to explore the activation of the PFC in children with different subtypes of ADHD during executive function task.

**Methods:**

Participants in this study include typically developing (TD) children (*n* = 28), ADHD-predominantly inattentive (ADHD-PI) (*n* = 39) and ADHD-combined (ADHD-C) (*n* = 24). To examine the executive function of ADHD, the Go/No-go task is chosen to assess the response inhibition function. The activation of PFC in all participants during the Go/No-go task was recorded by fNIRS. Meanwhile, behavioral data were also recorded.

**Results:**

Both TD and ADHD children activated the right PFC [middle frontal gyrus (MFG) and inferior frontal gyrus (IFG)] during response inhibition. However, the range and degree of activation differed among these groups. Compared with TD children, those with ADHD-PI had a smaller extent of activation in the right PFC, and those with ADHD-C only had a tendency to enhance activation. In addition, children with ADHD-PI and ADHD-C had impaired activation of the temporal gyrus. Besides, compared with ADHD-C and TD, those with ADHD-PI also had impaired activation of the right precentral gyrus (PG), and the supplementary motor area (SMA). Compared with ADHD-PI, ADHD-C showed decreased activation of the right MFG. The activation of Ch34 (BA44, rPFC) in children with ADHD-PI and ADHD-C was negatively correlated with their clinical symptoms.

**Conclusion:**

The activation of the PFC in children with different subtypes of ADHD has both commonalities and differences. The degree of activation of the right PFC Ch34 in children with ADHD is negatively correlated with clinical symptoms. fNIRS could be served as a candidate hemodynamic biomarker for the diagnosis of ADHD.

## 1. Introduction

Attention deficit hyperactivity disorder (ADHD) is a common chronic neurodevelopmental disorder in children and adolescents that affect approximately 7.2% of children worldwide (Thomas et al., 2015). Around 60–66% of children with ADHD have at least one comorbidity, such as sleep disturbance, tic disorder, and oppositional defiant disorder (Thapar and Cooper, 2016). The symptoms and comorbidities of ADHD may lead to problems in education, family, and social interactions (Reale et al., 2017). According to clinical symptoms, ADHD is divided into three subtypes/presentations: predominantly inattentive (ADHD-PI), predominantly hyperactive-impulsive (ADHD-HI), and combined subtype (ADHD-C) (Hu, 2003; Francesmonneris et al., 2013).

Despite its complex and unknown etiology, ADHD is related to cognitive deficits, especially executive dysfunction. Barkley (1997) proposed that one of the core deficits in ADHD is the impairment of response inhibition in executive function, in which the prefrontal cortex (PFC) plays an important role (Schmitz et al., 2006; Kana et al., 2007).

Children with different subtypes of ADHD differ not only in clinical presentation, comorbidities, and response to treatment, but also in pathogenesis (Willcutt et al., 2012; de la Peña et al., 2020). Children with ADHD-PI have greater activation in temporal and parietal regions and bilateral middle frontal gyrus than those with ADHD-C. Children with ADHD-C have greater activation in the bilateral medial occipital lobes than those with ADHD-PI (Solanto et al., 2009). fMRI is preferred for brain function research due to its high spatial resolution. However, head movement and noise limit its application in children.

Functional near-infrared spectroscopy (fNIRS) is a non-invasive optical brain function detection technique that can indirectly detect changes in oxygenated hemoglobin (oxy-Hb) and deoxygenated hemoglobin (deoxy-Hb) concentrations in the cerebral cortex over time. fNIRS has several advantages over fMRI, including insensitivity to head movement, cost-effectiveness, portability, and high time resolution (Gossé et al., 2021). In addition, the portion of the PFC that was damaged in children with ADHD can be detected. Therefore, this method is particularly suitable for children with ADHD who are poorly coordinated. An increasing number of studies applied fNIRS to explore executive dysfunction in children with ADHD. Researchers used fNIRS to investigate PFC activation during a response inhibition task (Go/No-go) in children with ADHD. The results showed that PFC activation was significantly lower in children with ADHD than in typically developing (TD) children, but the location of the reduced activation varied across studies. In these reports, hypoactivity in the right frontal lobe was the most common, including right PFC, right inferior frontal gyrus (rIFG), and right middle frontal gyrus (rMFG) (Inoue et al., 2012; Monden et al., 2012, 2015; Xiao et al., 2012; Nagashima et al., 2014). Reduced activation was also observed in the left frontopolar cortex (Miao et al., 2017) or bilateral PFC (Bell et al., 2020). These differences may be related to the small sample size, the varying ages and medications of the enrolled children, and the lack of ADHD typing.

No research was conducted on response inhibition in different ADHD subtypes by using fNIRS. In the current study, fNIRS was utilized to explore the activation of the PFC during response inhibition (Go/No-go task) in children with different ADHD subtypes (ADHD-PI and ADHD-C). ADHD-H was excluded due to its small population and poor cooperation. We hypothesized that ADHD-PI and ADHD-C differ in their response inhibition function and cortical activation levels.

## 2. Materials and methods

### 2.1. Participants

63 children with ADHD and 28 typically developing children (gender: 14 boys and 14 girls, mean age: 8.07 ± 1.61 years) were recruited from the Children’s Hospital affiliated to the Capital Institute of Pediatrics in China. Among the children with ADHD, 39 had ADHD-PI (gender: 28 boys and 11 girls, mean age: 8.38 ± 1.54 years) and 24 had ADHD-C (gender: 21 boys and 3 girls, mean age: 7.75 ± 1.36 years). Demographic information is shown in Table 1.

**TABLE 1:** Demographic information for ADHD children and TD children.

	TD children		ADHD-PI		ADHD-C		χ^2^/F	*p*
	Mean	SD	Mean	SD	Mean	SD		
Gender (boy/girl)	1:1		28:11		7:1		8.742	0.013*
Age	8.07	1.61	8.38	1.54	7.75	1.36	1.296	0.279^ns^

SD, standard deviation; χ^2^, Chi-squared; F, F value; *p*, *p* value. Statistical significances are presented as follows: *p < 0.05; and ns, not significant.

Inclusion criteria for ADHD were as follows: ➀ 6–12 years old; ➁ ADHD diagnosed according to the Diagnostic and Statistical Manual of Mental Disorders, Fifth Edition (DSM-5); ➂ no pharmacological and non-pharmacological treatment of ADHD; ➃ IQ ≥ 80 on the Chinese version of Wechsler Intelligence Scale for Children Revised (WISC-CR); ➄ right-handedness; ➅ good experimental cooperation; and ➆ no chronic physical illness and psychiatric disorders. All parents of children with ADHD completed the Chinese version of the Swanson, Nolan, and Pelham Questionnaire (SNAP-IV, 9 items for attention deficit, 9 items for hyperactivity) and the ADHD Rating Scale IV (ADHD-RS-IV).

Inclusion criteria for TD children were as follows: ➀ 6–12 years old; ➁ IQ ≥ 80 on the Chinese version of WISC-CR; ➂ right-handedness; ➃ good experimental cooperation; and ➄ no chronic physical illness and psychiatric disorders.

### 2.2. Experimental task

The Go/No-go task in this study was similar to that used by Miao et al. (2017), Monden et al. (2012), and Monden et al. (2015). The task was presented on a computer monitor 50 cm away from the subject and divided into six block sets, each of which contains a Go block (baseline task) and a Go/No-go block (target). Every Go or Go/No-go block comprises 24 stimuli, each appearing as a cartoon picture in the center of the computer screen and lasted for 300 ms. The interval between stimuli was 700 ms. Each block was preceded by a 3-second tutorial. The duration of a block set was 54 s, and the duration of the whole task was 324 s.

**FIGURE 1:**
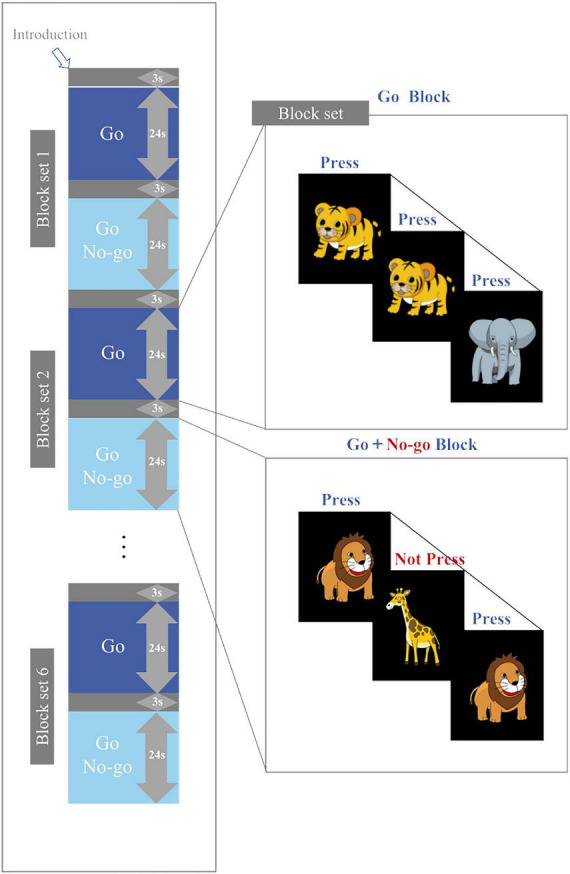
Task design.

In the Go block, a random sequence of “elephant” and “tiger” pictures was presented on the screen. Subjects were asked to press a button as soon as they saw the “elephant” and “tiger”. In the Go/No-go block, a random sequence of “lion” and “giraffe” pictures was presented on the screen (lion: giraffe = 1:1), as shown in Figure 1. The subjects were asked to press a button quickly when they saw the “lion” and not to press the button if they saw the “giraffe”. All subjects pressed the button with their right index finger. Prior to the test, the subjects underwent a practice session to ensure that they understood the procedure. Brain activity was measured with fNIRS while the subjects performed the Go/No-go task. The accuracy and reaction time of the task were also recorded.

### 2.3. fNIRS measurements

This study used multi-channel fNIRS (ETG-4000; Hitachi, Japan) utilizing two wavelengths of near-infrared light, namely, 695 and 830 nm. Detection depth was 20–30 nm below the scalp, and collecting frequency was 10 Hz. Changes in near-infrared (NIR) light absorption through the cerebral cortex and the modified Beer-Lambert law were calculated to indirectly determine the concentrations of oxyhemoglobin and deoxyhemoglobin in the cerebral cortex under 10 Hz collecting frequency (Cope et al., 1988; Maki et al., 1995).

**FIGURE 2:**
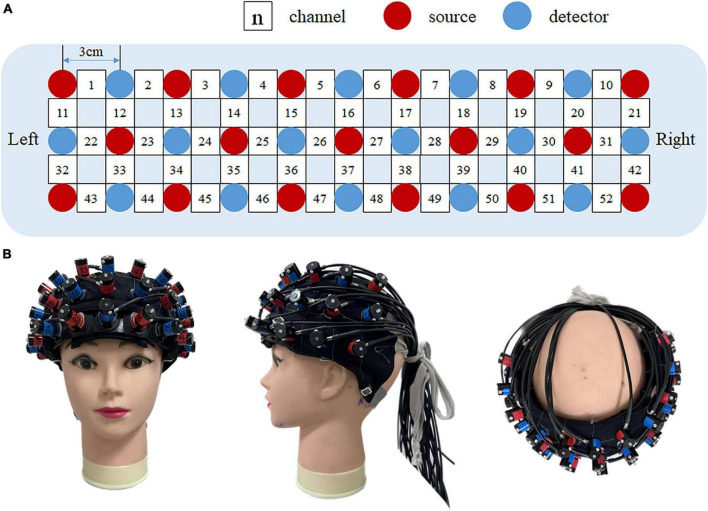
fNIRS measurement setting. **(A)** The location of 52 channels and 33 probes. Red dots indicate 17 fNIRS sources, blue dots indicate16 fNIRS detectors, and white squares indicate 52 fNIRS channels. **(B)** Position of the optode plate on the subject’s forehead. Left to right: anterior view, side view, and top view, respectively.

In brief, 33 probes (17 sources and 16 detectors) were placed in the subject’s prefrontal lobe through a 3 × 11 probe holder. A channel was formed between two adjacent probes (spaced 3 cm apart) to obtain a total of 52 channels (Figure 2). The probes were placed according to the International EEG 10–20 system. The lowermost probe in the middlemost row was placed in FPz firstly, and the remaining channels in the lowermost row were oriented along the brow arch and T3/T4. Head circumference (HC), biparietal diameter (BD), and occipitofrontal diameter (OA) were measured in all enrolled children, and the mean values were calculated. The purpose of the measurements was to confirm whether the channels covered our target brain regions. Children whose head size is roughly in line with these three averages were selected for localization. A transcranial neuronavigation system (State Key Laboratory of Cognitive Neuroscience, Beijing Normal University) was used to locate the positions of 52 channels (Xiao et al., 2018; Zhang et al., 2021). This system performs cranial shell model reconstruction based on the locations of reference points on the subject’s head, including central midline (Cz), inion (Iz), nasion (Nz), right anterior ear (AR), and left anterior ear (AL). The location of the channel was then determined from the transcranial brain atlas database.

### 2.4. Data analysis

This work adopted a multilevel model analysis of oxyhemoglobin, which is more sensitive to changes in cerebral blood flow than deoxyhemoglobin (Strangman et al., 2002; Hoshi, 2003). HOMER2 (Huppert et al., 2009) and NIRS-SPM v4.1 (Ye et al., 2009) were utilized to preprocess the fNIRS data. HOMER2 was used to remove the noise of fNIRS channels and transfer the optical intensity to the optical density (OD). The NIRS-SPM was used to calculate the concentration changes of oxy-Hb based on the modified Beer-Lambert law (Cope and Delpy, 1988) and remove the baseline drift with the wavelet detrending procedure. To remove the high-frequency noise and the intrinsic temporal correlations, temporal smooth was applied by the hemodynamic response function (HRF) (Worsley and Friston, 1995).

At the first level, fNIRS data were analyzed using General Linear Models. The beta (β) values representing the amplitude of the task response were obtained based on the hemodynamic response modeling of the fNIRS channel. At the second level, a one-sample t-test (test value of 0) was performed on the beta (β) values. Differences were considered significant when *p* < 0.05, and test results were corrected with the FDR method. Paired t-test was applied to compare differences in cortical activation between the Go/No-go block and the Go block. One-way ANOVA was employed to compare between-group differences in cortical activation during Go/No-go task in ADHD-PI, ADHD-C, and TD children. Data from behavior were also assessed by one-way ANOVA.

Correlations between cortical activation and clinical scales in children with ADHD were analyzed using Spearman’s correlation. Intergroup gender distribution was analyzed with chi-square test, the other intergroup differences were analyzed by one-way ANOVA with *post hoc* test.

## 3. Results

### 3.1. Demographic information

Significant difference in gender (chi-square test, χ^2^ = 8.742, and *p* = 0.013) but not in age (one-way ANOVA, *F* = 1.296, and *p* = 0.279) was found among the subjects (Table 1).

### 3.2. Positioning of channels

The head size of the model (HC: 52 cm, BD: 31 cm, OA: 29 cm) is roughly in line with the average head circumference (52.21 ± 1.86 cm), biparietal diameter (30.97 ± 1.60 cm) and occipitofrontal diameter (28.73 ± 1.69 cm) of the children enrolled in the group.

The MNI coordinates and the maximum occupied brain area of each channel coded in a macro anatomical brain atlas LBPA40 (Shattuck et al., 2008) and Brodmann’s area (BA) (Rorden and Brett, 2000) are shown in Table 2. The results showed that the probe holder covered the regions of interest, i.e., PFC.

**TABLE 2:** Estimated locations of the 52 fNIRS channels.

Channel	MNI				BA (%)	LPBA (%)
	X (mm)	Y (mm)	Z (mm)	SD (mm)		
1	61.20	-29.10	49.43	4.83	40 (89%)	54 (90%)
2	56.11	-4.74	47.42	6.08	4 (55%)	47 (63%)
3	45.67	20.79	44.10	5.50	6 (68%)	41 (99%)
4	30.82	38.75	42.75	5.32	8 (100%)	41 (96%)
5	11.71	47.60	44.54	4.94	8 (93%)	50 (94%)
6	-8.53	47.95	45.15	4.88	8 (93%)	23 (97%)
7	-26.35	41.60	41.19	5.51	8 (100%)	14 (84%)
8	-42.35	26.16	43.18	5.12	8 (86%)	14 (98%)
9	-53.38	4.32	43.78	6.43	6 (61%)	20 (68%)
10	-58.64	-21.79	49.34	5.73	2 (68%)	19 (69%)
11	65.21	-41.36	36.41	4.85	40 (100%)	54 (62%)
12	66.24	-12.77	33.42	5.62	348%)	46 (61%)
13	59.14	9.06	32.21	5.38	6 (73%)	47 (84%)
14	44.88	34.03	34.21	5.35	8 (64%)	41 (98%)
15	24.13	55.65	29.57	5.05	968%)	41 (90%)
16	6.78	57.84	33.09	6.32	8 (73%)	50 (96%)
17	-20.05	54.01	32.66	4.94	8 (55%)	14 (80%)
18	-38.41	44.29	29.40	5.03	9 (100%)	14 (98%)
19	-51.76	22.28	32.52	5.63	9 (91%)	14 (79%)
20	-63.69	-5.08	33.22	5.12	4 (50%)	19 (73%)
21	-65.57	-34.02	38.29	4.94	40 (100%)	27 (94%)
22	68.37	-29.88	25.34	5.48	40 (89%)	54 (82%)
23	64.62	4.59	20.10	5.50	6 (48%)	47 (77%)
24	53.53	32.30	17.56	5.09	46 (68%)	35 (86%)
25	35.75	57.21	14.97	5.02	10 (89%)	41 (92%)
26	13.96	65.59	19.28	4.80	9 (89%)	50 (52%)
27	-13.06	65.49	19.50	4.54	9 (91%)	23 (64%)
28	-33.56	54.86	20.58	4.96	9 (75%)	14 (97%)
29	-49.17	38.31	18.51	5.25	46 (91%)	14 (59%)
30	-62.05	8.96	17.90	6.07	6 (45%)	20 (66%)
31	-68.22	-17.47	22.67	6.04	40 (34%)	19 (61%)
32	68.60	-40.31	12.06	5.66	22 (95%)	53 (59%)
33	67.47	-7.62	9.92	6.60	43 (45%)	46 (58%)
34	59.93	16.49	13.29	6.49	44 (57%)	35 (70%)
35	46.42	46.71	11.85	5.50	10 (57%)	35 (58%)
36	24.32	64.68	12.09	5.21	10 (95%)	41 (97%)
37	7.22	68.93	9.97	5.53	10 (98%)	50 (93%)
38	-21.63	65.67	12.54	4.49	10 (100%)	14 (94%)
39	-39.38	55.90	7.13	4.57	10 (86%)	14 (60%)
40	-55.01	32.36	6.90	4.88	46 (82%)	8 (99%)
41	-64.69	-2.49	5.76	7.28	22 (36%)	19 (34%)
42	-69.51	-29.81	8.19	5.38	42 (52%)	26 (88%)
43	70.10	-22.36	-6.81	5.52	21 (84%)	44 (68%)
44	59.18	8.68	-14.72	7.55	38 (36%)	53 (59%)
45	53.23	36.57	-3.42	5.24	45 (75%)	35 (97%)
46	37.38	59.16	-2.14	4.02	10 (100%)	35 (66%)
47	15.39	69.28	-1.23	4.35	10 (100%)	41 (67%)
48	-11.14	69.11	-3.88	4.04	10 (100%)	23 (85%)
49	-33.83	61.54	-2.33	3.83	10 (100%)	14 (60%)
50	-49.04	43.72	-6.16	3.96	46 (48%)	8 (95%)
51	-58.14	10.98	-9.09	9.56	22 (50%)	26 (65%)
52	-68.76	-13.03	-13.36	5.30	21 (98%)	17 (82%)

Each channel shows the mean and standard deviation (SD) of each channel’s MNI coordinates and the maximum occupied brain area coded in a macro anatomical brain atlas LBPA40 (Shattuck et al., 2008) and Brodmann’s area (BA) (Rorden and Brett, 2000). The percentage of channels in that brain region is shown in parentheses.

### 3.3. Behavioral data

Table 3 summarizes the behavioral data during the Go/No-go task, including reaction time (RT) and accuracy (ACC) for TD children and those with ADHD-PI and ADHD-C. One-way ANOVA results showed no significant differences in reaction time and accuracy during Go blocks and in response time during Go/No-go blocks among the three groups. Accuracy for the TD children group during Go/No-go blocks was significantly higher than that for the ADHD-C group (*p* < 0.05).

**TABLE 3:** Behavioral data and fNIRS data associated with response inhibition during go/no-go task.

	1. TD children (*N* = 28)	2. ADHD-PI (*N* = 39)	3. ADHD-C (*N* = 24)	*F*	*p*	*Post-hoc* test
**Behavioral data**						
Go block RT (ms)	377.532 ± 81.564	354.222 ± 67.865	368.822 ± 62.221	0.920	0.402^ns^	
Go/No-go block RT (ms)	453.394 ± 57.279	449.009 ± 69.490	452.490 ± 47.737	0.049	0.952^ns^	
Go block ACC (%)	85.044 ± 12.422	86.388 ± 8.603	83.825 ± 12.511	0.413	0.663^ns^	
Go/No-go block ACC (%)	90.129 ± 7.976	85.986 ± 9.422	82.755 ± 9.193	4.471	0.014*	1 vs. 3*
**fNIRS data**						
Ch4	0.0083 ± 0.544	0.0203 ± 0.6406	−0.0384 ± 0.1261	3.380	0.039*	2 vs. 3*
Ch23	0.0123 ± 0.054	−0.0389 ± 0.1102	0.0096 ± 0.0554	3.734	0.028*	1 vs. 2* 2 vs. 3*
Ch34	0.0271 ± 0.407	−0.0118 ± 0.7001	−0.0061 ± 0.6839	3.353	0.040*	1 vs. 2*
Ch44	0.4495 ± 0.1076	−0.0133 ± 0.0986	−0.0266 ± 0.1065	3.537	0.034*	1 vs. 2* 1 vs. 3*
Ch51	0.0441 ± 0.0879	−0.0057 ± 0.1132	−0.0211 ± 0.0748	3.218	0.045*	1 vs. 3*
Ch52	0.0518 ± 0.0890	−0.0188 ± 0.0854	−0.0243 ± 0.0780	6.599	0.002**	1 vs.2** 1 vs.3**

Data are presented as mean ± SD. TD children, typical development; ADHD-PI, ADHD-predominantly inattentive; ADHD-C, ADHD-combined; RT, reaction time; ACC, accuracy; Ch, channel. *p < 0.05; **p < 0.01; ns, not significant, *p* > 0.05.

### 3.4. fNIRS data

**FIGURE 3:**
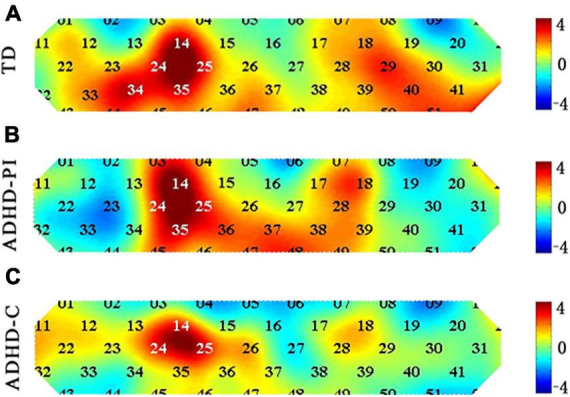
Heat map of *t*-values comparing the activation of TD children **(A)** and those with ADHD-PI **(B)** and ADHD-C **(C)** during response inhibition (Go/No-go block minus Go block) for 52 channels. The 52 channels are colored by their t values according to the color scale. As shown in the color scale on the right, warm colors represent positive *t*-values, and cool colors represent negative *t*-values. A dark color indicates a large absolute *t*-value.

TD children and those with ADHD-PI and ADHD-C showed differences in the channels activated during response inhibition (Go/No-go block minus Go block). The activation of channels 14 (*t* = 3.713, *p-corrected* = 0.024), 25 (*t* = 3.773, *p-corrected* = 0.041), 34 (*t* = 3.467, *p-corrected* = 0.032), and 35 (*t* = 3.448, *p-corrected* = 0.024) of TD children was significantly enhanced. The activation of channels 14 (*t* = 4.530, *p-corrected* = 0.003), 25 (*t* = 3.645, *p-corrected* = 0.021), and 35 (*t* = 3.615, *p-corrected* = 0.016) of children with ADHD-PI was significantly enhanced. A trend of enhanced activation was observed for channel 25 (*t* = 3.688, *p-corrected* = 0.067) of ADHD-C, but this result was not significant (0.1 > *p* > 0.05). Figure 3 shows the heat maps of the t values.

The results of between-group ANOVA showed that the activation of channel 23, 34, 44, and 52 in children with ADHD-PI was significantly lower than that in TD children (*p* < 0.05). The activation of channel 44, 51, and 52 in children with ADHD-C was significantly diminished compared with that in TD children (*p* < 0.05). Compared with ADHD-C, ADHD-PI showed increased activation of channel 4 and decreased activation of channel 23 (Table 3). The distribution of activation for three groups in CH 4, 23, 34, 44, 51, 52 is shown in Supplementary Figure 1.

### 3.5. Correlation

Table 4 shows the Spearman’s correlations between the behavior scale (SNAP-IV and ADHD-RS- IV) and the oxy-Hb changes at four channels (Ch14, Ch25, Ch34, and Ch35) activated during the Go/No-go task. The activation of Ch34 in ADHD-PI was negatively correlated with the inattentive symptom severity scores of SNAP-IV (*r* = 0.396, *p* = 0.017). The activation of Ch34 in ADHD-C was negatively correlated with the total score of ADHD-RS- IV (*r* = −0.482, *p* = 0.027).

**TABLE 4:** The correlation between scale score and channel activation.

		ADHD-PI				ADHD-C			
		Ch14	Ch25	Ch34	Ch35	Ch14	Ch25	Ch34	Ch35
SNAP-IV	Attention deficit	−0.09	−0.063	−0.396*	0.073	0.073	0.283	0.157	0.386
	Hyperactive	0.014	−0.004	−0.08	−0.053	0.266	0.403	−0.205	0.325
ADHD-RS-IV		−0.071	−0.162	−0.118	−0.074	−0.227	−0.127	−0.482*	−0.025

*p < 0.05.

## 4. Discussion

This study used fNIRS to explore the similarities and differences of cortical hemodynamics among TD children and those with ADHD-PI and ADHD-C. The right PFC is the core brain region for response inhibition, and the range of PFC activation on the right side of ADHD-PI and ADHD-C is reduced compared with that in TD children. The activation of the PFC in children with different subtypes of ADHD has both commonalities and differences.

### 4.1. fNIRS

In the fNIRS analysis, the contrast of Go/No-go block against Go block was adopted. Previous fMRI studies of response inhibition have consistently revealed the activation of the frontal lobes (Simmonds et al., 2008). Therefore, the prefrontal lobe served as the main target brain region in this study. Results showed that the activation of right MFG and right IFG (BA8/10/44) was significantly enhanced in TD children during the Go/No-go task. The site of activation in children with ADHD was similar to that in TD children, but the extent of activation was reduced. The activation of right MFG and right IFG (BA8/10) was significantly enhanced in children with ADHD-PI. But the activation of right IFG in children with ADHD-PI was significantly lower than that in TD. ADHD-C had only a trend of enhanced activation of the right MFG (BA10).

This finding is consistent with the majority of fNIRS studies (Monden et al., 2012, 2015; Xiao et al., 2012; Nagashima et al., 2014), which all suggested that the right side of the IFG and MFG function abnormally during Go/No-go in ADHD. However, some findings showed the reduced activity in the left and right PFC (Bell et al., 2020), left frontopolar cortex (Bell et al., 2020), or right lateral PFC (Kaga et al., 2020) in ADHD. These differences may be related to the proportion of subtypes of ADHD in the participants. The current results do not support this view. Despite the lack of information on ADHD-HI, the main sites of damage in ADHD-PI and ADHD-C are the right IFG and MFG. The difference in results may be related to the stimulus format of the Go/No-go task. With a few exceptions (Xiao et al., 2012; Nagashima et al., 2014), most of the studies with impaired right-sided activation used picture stimuli (including the present study) and most of those focusing on impaired left-sided activation were stimulated by arrows in different directions and letter (Smith et al., 2006; Rubia et al., 2009; Cubillo et al., 2011; Miao et al., 2017; Bell et al., 2020). Despite individual differences in cognitive style, the right brain predominantly performs picture and spatial processing, and the left brain dominates language processing (Levy, 1974; Vogel et al., 2003; Krumbholz et al., 2007). Therefore, the type of stimulus could be an important influencing factor. In addition, the age of the enrolled children, sample size, medication, instrumentation, and wavelength of NIR light may have influenced the study results. Standardized experimental design and enrollment criteria are needed in future works.

This study showed that the activation of the left middle temporal gyrus (BA21) and the right superior temporal gyrus (BA38) was significantly reduced both in children with ADHD-PI and ADHD-C compared with that in TD children. A meta-analysis showed that the ventral attentional network and frontoparietal network are reduced in ADHD (Cortese et al., 2012). The middle temporal gyrus is related to the ventral attentional network and plays an important role in recognition, memory, auditory processing, and language comprehension (Bialystok et al., 2012; Whipple and Nelson, 2016; Craik, 2020). Animal studies also revealed that damaged temporal lobe impairs visuospatial working memory and shows increased activity and impulsivity (Ramos et al., 2016). This finding suggested that the impaired response inhibition in ADHD is associated with the temporal lobe.

The activation of the right precentral gyrus (PG) and supplementary motor area (BA6) was impaired in children with ADHD-PI compared with that in TD and ADHD-C subjects. The precentral gyrus (PG) consists of the primary motor cortex and plays a critical role in the final processing phase of response inhibition (Stinear et al., 2009). The supplementary motor area (SMA) activates during response inhibition, and its dysfunction may lead to hyperactivity in ADHD (Karl et al., 2006). A fMRI meta-analysis showed significantly lower activation of PG and SMA during response inhibition in ADHD compared with that in controls (Lei et al., 2015). Daniel et al. suggested that the activation of pre-SMA is essential for response inhibition (Simmonds et al., 2008). The differences in behavioral performance and activation of PG and SMA during response inhibition in children with ADHD-PI and ADHD-C may be related to phenotypic differences between subtypes. However, Marion et al. suggested that the hemodynamic response to No-go signals is a combination of cognitive processes, and the activation of pre-SMA is driven by heightened attention or working memory rather than by response inhibition processes (Criaud and Boulinguez, 2013).

### 4.2. Correlation

Spearman’s correlations showed that oxy-Hb changes in Ch34 (right inferior frontal gyrus, BA44) negatively correlated with attention deficit scores in ADHD-PI, and total ADHD-RS- IV scores in ADHD-C. This finding suggested that with low rPFC activation, the symptoms in children with ADHD become severe. The right inferior frontal gyrus may be the core brain region for response inhibition function in ADHD. Monden et al. used the border of inferior and middle frontal gyri to measure the activation levels that can distinguish between ADHD and TD. The area under the receiver operating characteristic curve was 0.85, and the sensitivity was 0.9 (Monden et al., 2015). In the future, ch34 could be considered as a target for auxiliary diagnosis and fNIRS neurofeedback to further explore and validate the role of rPFC in ADHD pathogenesis.

## 5. Limitation

This study has several limitations. First, ADHD-H was excluded due to its small population and poor cooperation. A comparison between ADHD-H and the other two subtypes was not performed. Second, the age span of participants was large. Frontal lobe function in children with ADHD changes with their age (Yasumura et al., 2019). Thirdly, short channels and continuous blood pressure monitoring was not conducted in this study, which could effectively remove the extracerebral, systemic components captured by fNIRS. In addition, the variation in the gender of participants might have influenced the fNIRS results. Owing to the limited sample size, gender-specific subgroup comparisons were not performed. Strict grouping methods, admission criteria and well-controlled experimental design are needed for future studies.

## 6. Conclusion

This study provides preliminary evidence on the similarities and differences in cortical hemodynamics among TD children and those with ADHD-PI and ADHD-C during response inhibition tasks. All three groups activated the right PFC (MFG and IFG) during response inhibition. However, the extent of activation differed among these groups. Compared with TD children, those with ADHD-PI had a smaller extent of activation in the right prefrontal lobe, and those with ADHD-C only had a tendency to enhance activation. In addition, children with ADHD-PI and ADHD-C had impaired activation of the temporal gyrus, and those with ADHD-PI also had impaired activation of the right PG and SMA. The activation of Ch34 (BA44, rPFC) in children with ADHD-PI and ADHD-C was negatively correlated with their clinical symptoms. These findings partly explain the phenotypic differences between ADHD-PI and ADHD-C.

## Data availability statement

The raw data supporting the conclusions of this article will be made available by the authors, without undue reservation.

## Ethics statement

The studies involving human participants were reviewed and approved by Ethics Committee of Capital Institute of Pediatrics. Written informed consent to participate in this study was provided by the participants or their legal guardian/next of kin.

## Author contributions

YZ: manuscript writing and data collection. SL, FZ, YR, JS, XW, and LW: data collection. TZ: data analysis. JY: experimental design and reviewing and revising the manuscript. All authors have read and agreed to the manuscript.
