# Supplementary material for: Response inhibition in children with different subtypes/presentations of attention deficit hyperactivity disorder: A near-infrared spectroscopy study
Source: Front Neurosci. 2023 Mar 2;17:1119289. doi: 10.3389/fnins.2023.1119289 (PMC10017865; doi:10.3389/fnins.2023.1119289)
Supplement: Supplementary file 1 [file Data_Sheet_1.docx]

Supplementary Material

# Supplementary Figures


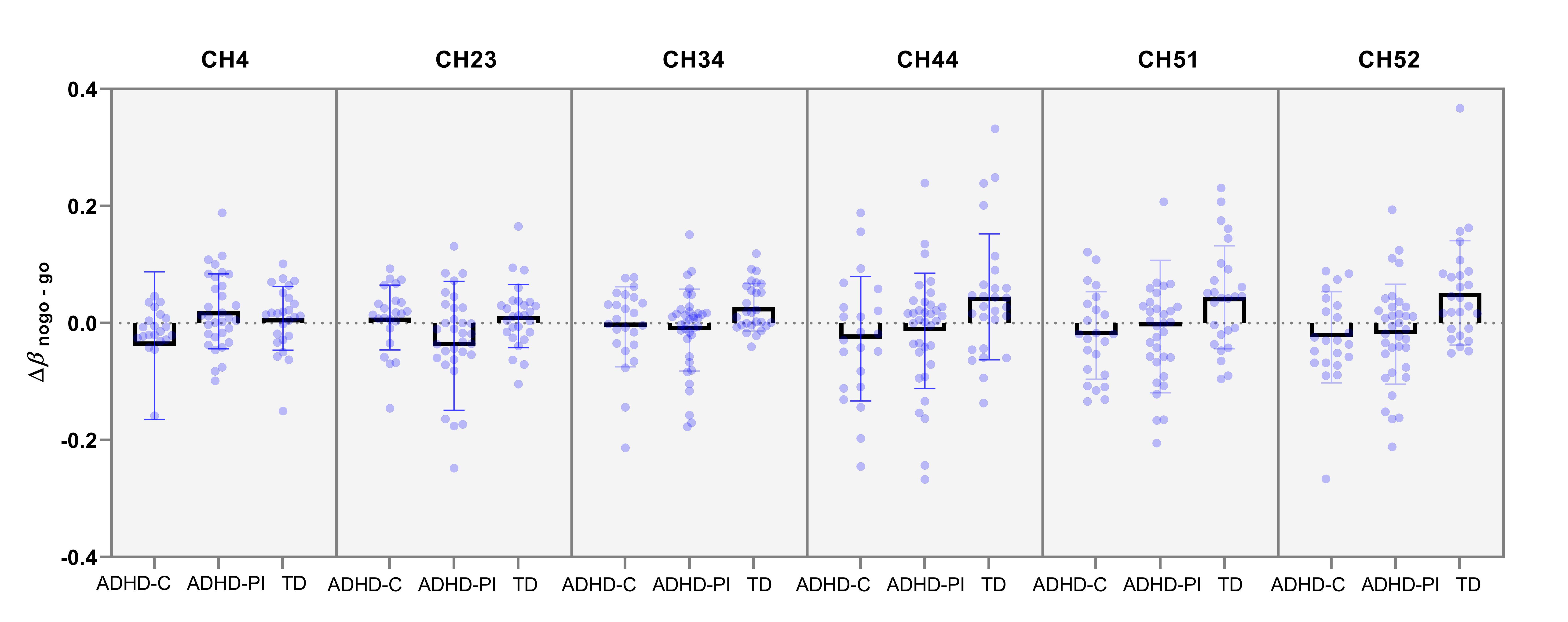


Figure S1.The distribution of activation (∆β_no-go_-∆β_go_) for three groups in CH 4, 23, 34, 44, 51, 52. The black box indicates the mean. Vertical lines represent the standard error (SD) of activity.
